# Development of Magnesium Aluminate (MgAl_2_O_4_) Nanoparticles for refractory crucible application

**DOI:** 10.1371/journal.pone.0296793

**Published:** 2024-01-16

**Authors:** Shaheer Ahmed Khan, Zakaria Mohd Zain, Ziauddin Siddiqui, Wajahat Khan, Abdul Aabid, Muneer Baig, Mohammad Abdul Malik

**Affiliations:** 1 Department of Engineering Sciences, Pakistan Navy Engineering College (PNEC), National University of Sciences and Technology (NUST), Karachi, Pakistan; 2 Department of Materials Engineering, NED University of Engineering and Technology, Karachi, Pakistan; 3 Department of Manufacturing & Materials Engineering, International Islamic University Malaysia (IIUM), Kuala Lumpur, Malaysia; 4 Department of Engineering Management, College of Engineering, Prince Sultan University, Riyadh, Saudi Arabia; UNICAMP, University of Campinas, BRAZIL

## Abstract

Ceramics are the oxides of metals and nonmetals with excellent compressive strength. Ceramics usually exhibit inert behavior at high temperatures. Magnesium aluminate (MgAl_2_O_4_), a member of the ceramic family, possesses a high working temperature up to 2000°C, low thermal conductivity, high strength even at elevated temperatures, and good corrosion resistance. Moreover, Magnesium Aluminate Nanoparticles (MANPs) can be used in the making of refractory crucible applications. This study focuses on the thermal behavior of Magnesium Aluminate Nanoparticles (MANPs) and their application in the making of refractory crucibles. The molten salt method is used to obtain MANPs. The presence of MANPs is seen by XRD peaks ranging from 66° to 67°. The determination of the smallest crystallite size of the sample is achieved by utilizing the Scherrer formula and is found to be 15.3 nm. The SEM micrographs provided further information, indicating an average particle size of 91.2 nm. At 600°C, DSC curves show that only 0.05 W/g heat flows into the material, and the TGA curve shows only 3% weight loss, which is prominent for thermal insulation applications. To investigate the thermal properties, crucibles of pure MANPs and the different compositions of MANPs and pure alumina are prepared. During the sintering, cracks appear on the crucible of pure magnesium aluminate. To explore the reason for crack development, tablets of MgAl_2_O_4_ are made and sintered at 1150°C. Ceramography shows the crack-free surfaces of all the tablets. Results confirm the thermal stability of MANPs at high temperatures and their suitability for melting crucible applications.

## 1. Introduction

Improvement and the development of the materials regarding their properties and specially the industrial applications have always been the great interest of scientists, engineers, and researchers. Search of the most efficient and economical materials is still being done even after the development of the advanced materials. Ceramics is one of the classes which is nonmetallic and inorganic materials and is indispensable in our daily life. Furthermore, engineered ceramics have received substantial focus because of their applications [1–3].

Spinel structure materials are represented by the general formula AB_2_X_4_ where A and B are the divalent and trivalent cations while X is typically chalcogens that have extensive applications nowadays due to their properties. Magnesium aluminate is a spinel structure ceramic material that has several advantages, including thermal stability at higher temperatures, lack of sensitivity to chemicals, insensitivity to heat, and an adsorptive surface [4, 5].

Magnesium aluminate spinel (MgAl_2_O_4_) is a well-established refractory material that has received significant attention over the years due to its unique properties. These properties include a good thermal conductivity, low thermal expansion coefficient at elevated temperatures, high melting point (2105°C), chemical inertness, and good chemical and mechanical strength. As a result of these advantageous characteristics, MgAl_2_O_4_ is suitable for application in various refractory applications [6, 7]. The manufacturing of high-quality MgAl_2_O_4_ powder is an important step in the application of MgAl_2_O_4_ spinel. The conventional approach for creating MgAl_2_O_4_ is a solid-state reaction of Al_2_O_3_ and MgO at around 1500°C. This technique is simple to implement and ideal for large-scale manufacturing [8], but the reaction temperature is quite high, and the end product lacks the requisite purity and particle size. In addition to the conventional method of synthesis of spinel MgAl_2_O_4_, molten salt synthesis is also used. In recent years, the molten salt synthesis (MSS) approach has received a lot of interest. In this process, the reaction medium is low melting salts, which allow reactants to combine in an atomic scale liquid fraction [9]. The MSS liquid/solid system enables more homogeneous mixing and diffusion of input materials than the conventional solid-state synthesis technique, resulting in a significant reduction of temperature and reaction time [10]. Safaei-Naeini et al. [11] employed the molten-salt approach to successfully produce MgAl_2_O_4_ nanoparticles at 850°C by heating various MgO- and Al_2_O_3_-containing precursors in KCl in stoichiometric proportions, nanosized spinel powders were created. Fazli et al. [12] used a molten-salt method to create nanocrystalline MgAl_2_O_4_ spinel. Nano alumina, magnesia, and lithium chloride were used as starting materials. The ideal sintering procedure for best outcomes was discovered as 850°C sintering temperature with a 3h soaking period. The optimal salt-to-oxide ratio has been stated to be 5:1. Zhang et al. [13] have described a molten synthesis process for Mg-Al spinel. The initial precursors were high-purity alumina and magnesia, and the solvent was KCl, which was utilized to investigate how varied salt concentrations influenced the shape. Zhang et al. [14] used molten-salt synthesis to create a MgAl_2_O_4_ (MA) spinel layer on a Ti_3_AlC_2_ substrate in a different study.

Magnesium aluminate spinel’s have been observed to possess a range of favorable features, making them highly utilized as refractory materials and structural materials in metallurgical applications [15]. Magnesium aluminate spinel, characterized by its diverse stoichiometric compositions, finds extensive utilization across a range of applications depending upon the specific environmental conditions of the intended application area. The primary utilization domains of magnesium aluminate spinel as refractory materials include cement rotary kilns, steel-teeming ladles [16–18], and the regenerators checker work element of glass tank furnaces [19, 20]. Spinel magnesium aluminate has been produced by numerous researchers, who have also suggested its potential application in the metallurgical industry [21]. Despite the existence of several refractory applications, there is a noticeable absence of literature regarding the crucible development of MgAl_2_O_4_ as it possesses highly desirable features that are essential for an efficient crucible, such as a high melting point, chemical inertness, high thermal stability, good chemical, and mechanical strength. Therefore, some efforts have been carried out in this paper to demonstrate the preparation of crucibles from Magnesium aluminate nanoparticles (MgAl_2_O_4_). Firstly, the synthesis of MgAl_2_O_4_ was conducted by using the molten salt method in different salt-to-oxide ratios. The resulting samples were characterized using SEM, XRD and Ceramography was also performed to investigate the surface of ceramic samples. Finally, a variety of crucibles were fabricated utilizing both pure MgAl_2_O_4_ and a combination of Al_2_O_3_.

## 2. Materials and methods

### 2.1. Supporting chemicals and equipment used in synthesis

Following are the chemicals and equipment used in the synthesis of Magnesium aluminate nanoparticles.

#### 2.1.1. Chemicals

High-quality reagents of lab grade were supplied by the laboratory. Magnesium Oxide Powder (MgO), and Aluminum Oxide Powder (Al_2_O_3_) were the raw materials used to prepare Magnesium Aluminate (MgAl_2_O_4_) nanoparticles. Potassium chloride (KCl) was used as the solvent.

### 2.2. Different methodologies used for preparing MgAl_2_O_4_ nanoparticles

**Fig 1:**
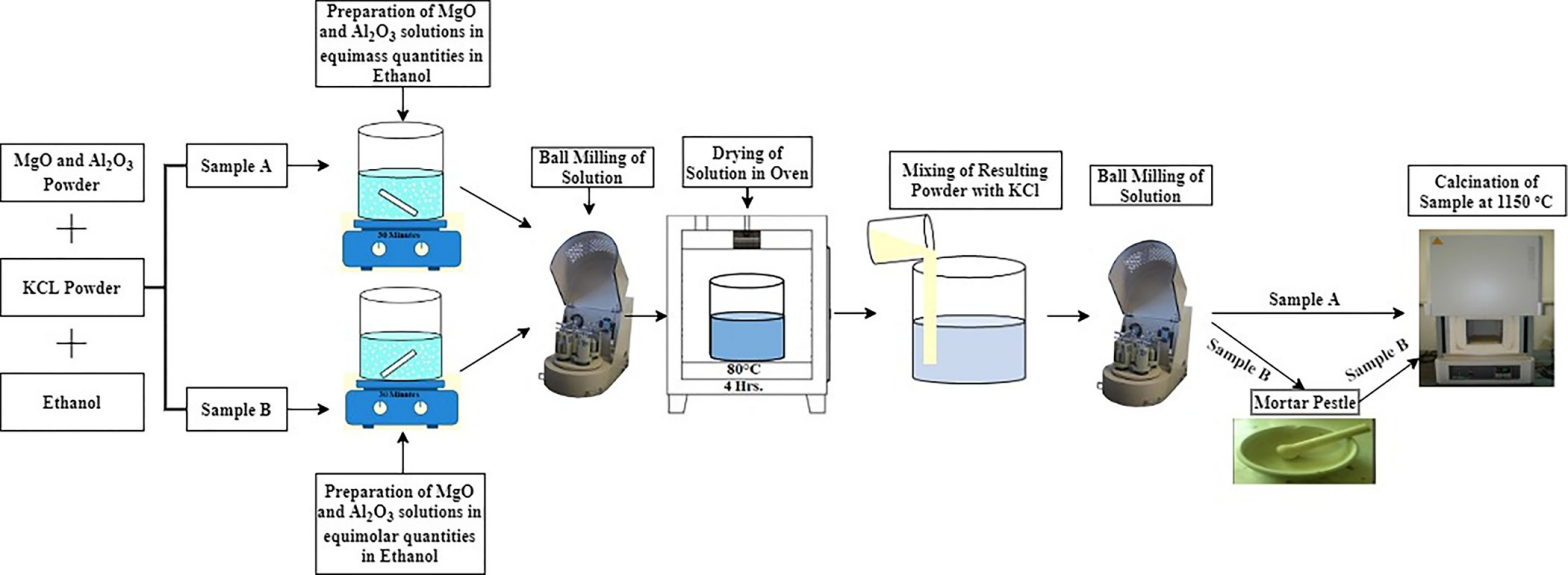
Different methodologies used for preparing MgAl_2_O_4_ nanoparticles.

As illustrated in Fig 1, two different samples were employed in the case of MgAl_2_O_4_. The process approach of Sample A was derived from the author’s own trial-and-error method. Sample A is a mixture of equimass quantities. In this method, precursors were prepared using a ratio of 15:1 (i.e., 15 g of KCl with 1g of MgO and an Al_2_O_3_ mixture). MgO and Al_2_O_3_ were then mixed together in equal amounts using ethanol as the solvent. In sample B, an equimolar mixture of MgO and Al_2_O_3_ is prepared in ethanol [13]. All methods remained consistent with Sample A, with the exception of an additional step involving the mixing of MgO and Al_2_O_3_ using a mortar and pestle for Sample B. The primary distinction between Sample A and Sample B lies in the composition of the mixtures, specifically the utilization of equimass and equimolar quantities, respectively.

The Experimentation Section provides a comprehensive discussion of the steps involved in each sample preparation.

### 2.3. Experimentation

**Fig 2:**
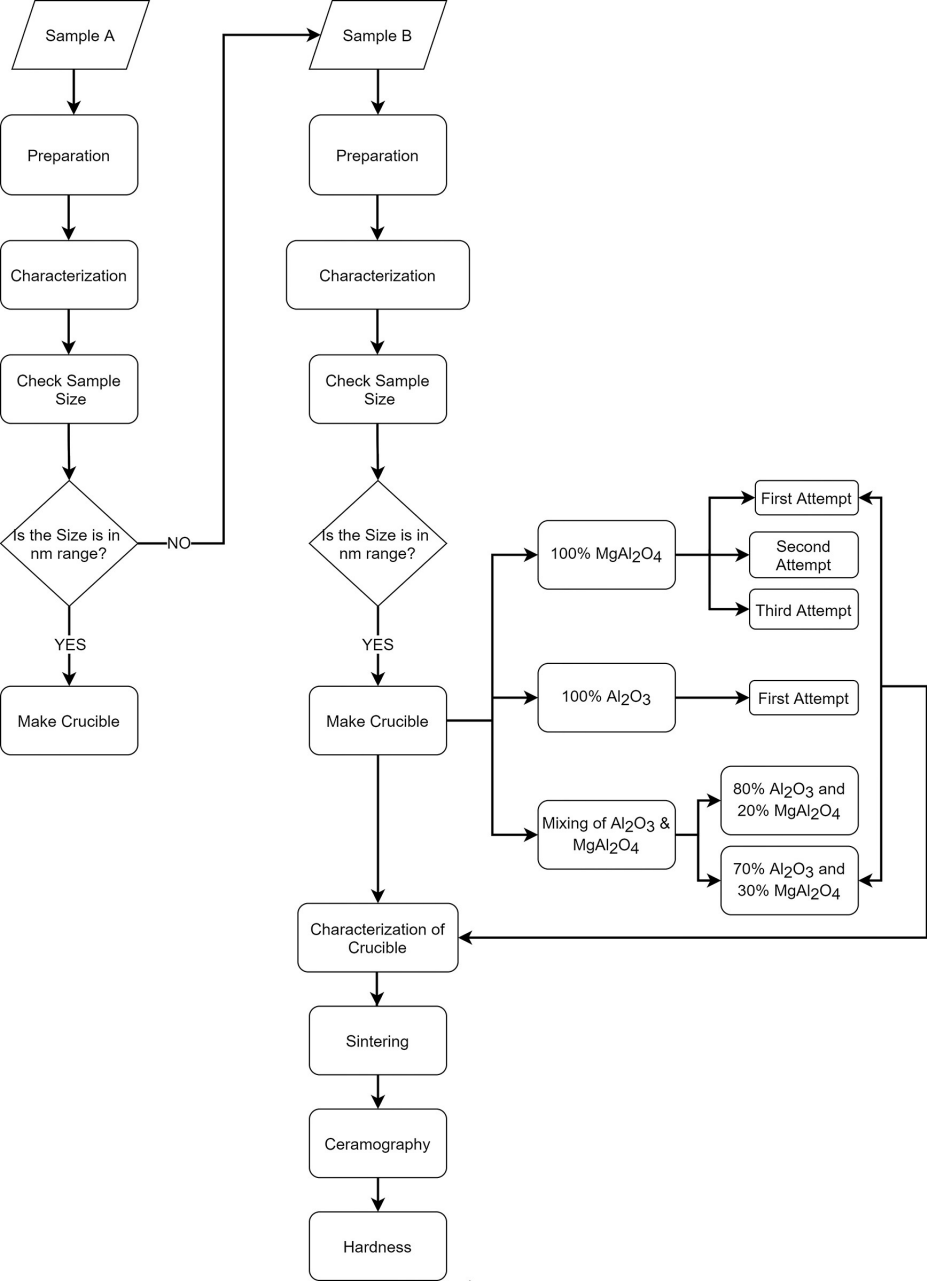
Experimentation plan.

A brief experimentation plan of the complete setup is shown in Fig 2.

#### 2.3.1. Preparation of Sample A

Magnetic stirrer was calibrated after placing the petri dish on the weight machine. 6g of fine Al_2_O_3_ powder was weighted and placed in a beaker. 6 g of MgO powder was weighted and placed with Al_2_O_3_ powder in the same beaker. 15ml of ethanol was added to the mixture with the help of a pipet. Then the beaker was placed on a magnetic stirrer for half an hour to mix the sample completely. After one hour, the powder settled at the bottom and excess ethanol drained out. The wet sample was placed in a ball milling machine for 3 hours. The sample was collected in the beaker and placed in a drying oven at 80°C for 4 hours after ball milling. The dried sample was then mixed with KCl powder. 30 g of KCl powder was mixed with 2g of dried sample (i.e. 15:1) and then placed in the ball milling machine for 1 hour.

After preheating the graphite crucible with its lid at 1150°C for 4 hours along with furnace cooling, the resulting sample was then placed in the preheated graphite crucible and tightly packed with its lid. The crucible was placed in a furnace for calcination at 1150°C for 4 hours. The sample was furnace cooled and ready for examination after calcination.

#### 2.3.2. Preparation of Sample B

1 M solutions of both MgO and Al_2_O_3_ were prepared by mixing 1 g of MgO powder in 25 ml of ethanol and 2.5g of Al_2_O_3_ powder in 25 ml of ethanol. Table 1 shows the details of Sample B solution preparation.

**Table 1:** Preparation of equimolar concentration solution.

Chemicals	Concentration (M)	Solute (g)	Solvent (ml)
MgO	1	40 4 1	1000 100 25
Al_2_O_3_	1	100 10 2.5	1000 100 25

The two solutions are then put in a beaker. The beaker was placed on a magnetic stirrer for half an hour in order to mix the solution completely. The beaker was covered with aluminum foil and placed in a fume hood for 22 hours. The powder settled down at the bottom and excess ethanol drained out. The wet sample was placed in a ball milling machine for 3 hours. After ball milling, the sample was collected into the beaker and placed in a drying oven at 80°C for 4 hours. 120 g of KCl powder is mixed with 4g of dried sample (i.e., 30:1) and placed in a ball milling machine for 1 hour. Then again, milled in a mortar pestle for 15 min. The resulting sample was then placed in a preheated graphite crucible and tightly packed with its lid. The crucible was placed in a furnace for calcination at 1150°C for 4 hours. The sample was furnace cooled and ready for examination after calcination.

### 2.4. Characterization

Characterization is an organized examination or formal evaluation exercise. It involves the measurements, tests, and gauges applied to certain characteristics regarding an object or activity. Table 2 shows the equipment used to characterize different characteristics of Magnesium aluminate nanoparticles.

**Table 2:** Characterization of MgAl_2_O_4_.

Machine/Technique	Purpose
Laser Particle Analyzer	Determination of average particle size of Sample A
X-Ray Diffraction	Examine the diffraction pattern of Sample B
Differential Scanning Calorimeter/Thermogravimetric Analysis	Thermal Analysis of Sample B
Stereo Microscope	Low Resolution surface investigation of Sample B
Metallurgical Microscope	High Resolution surface investigation of Sample B
Scanning Electron Microscope	Morphological analysis of the Sample B
Rockwell Hardness Tester	Hardness calculation of Sample B

## 3. Results and discussion

### 3.1. Result of Sample A

**Fig 3:**
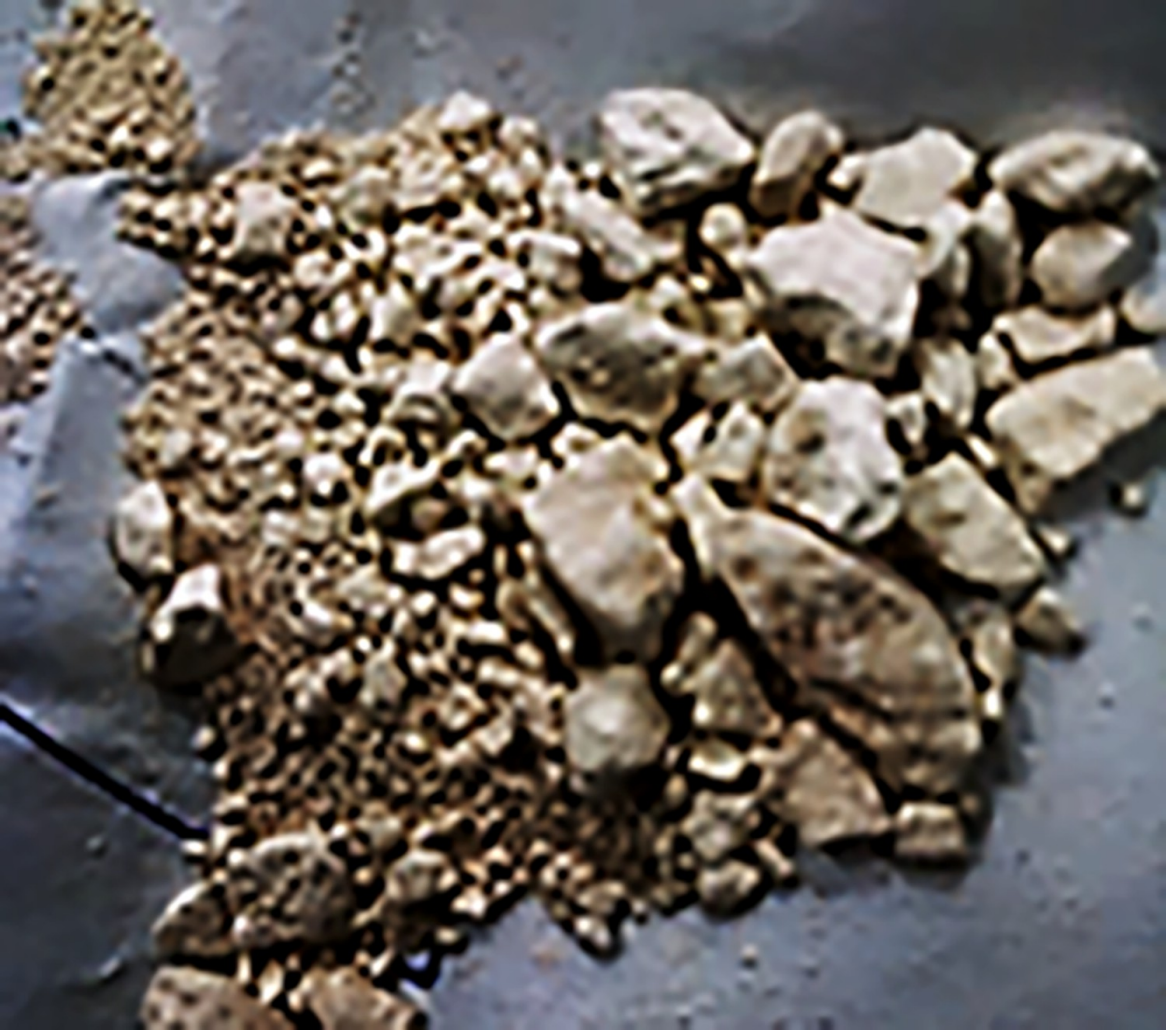
Sample A.

**Fig 4:**
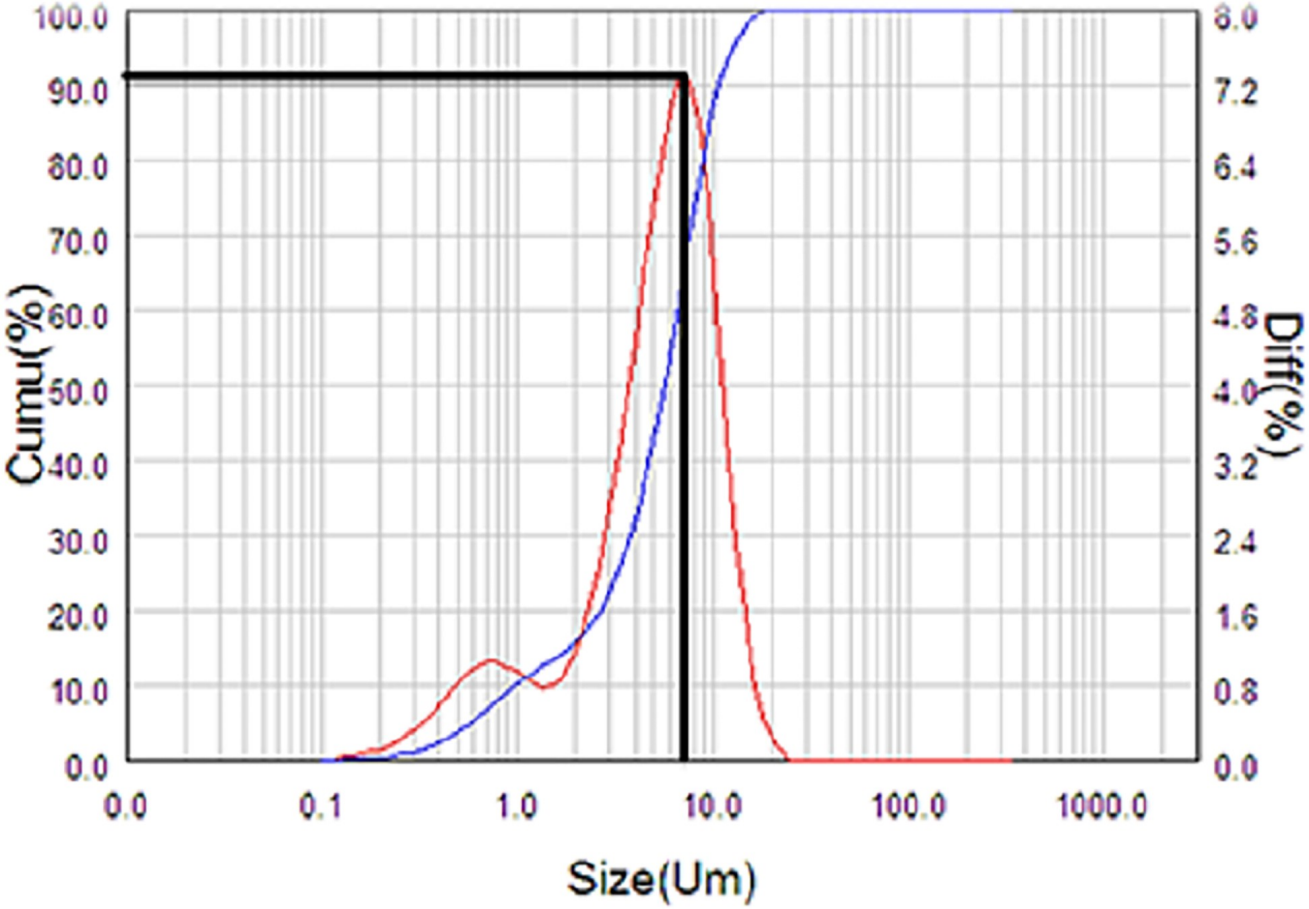
Graph showing the particle size of Sample A.

Laser Particle Size Analyzer was used to obtain the average particle size. Fig 3 depicts the resultant sample A. The graph in Fig 4 shows that the particle size was 6 μm and was not considered in the nano range (<100 nm). This is the case of mixing the quantities of magnesium oxide and aluminum oxide in equimass quantities rather than equimolar quantities, as stated in [13].

### 3.2. Results of Sample B

**Fig 5:**
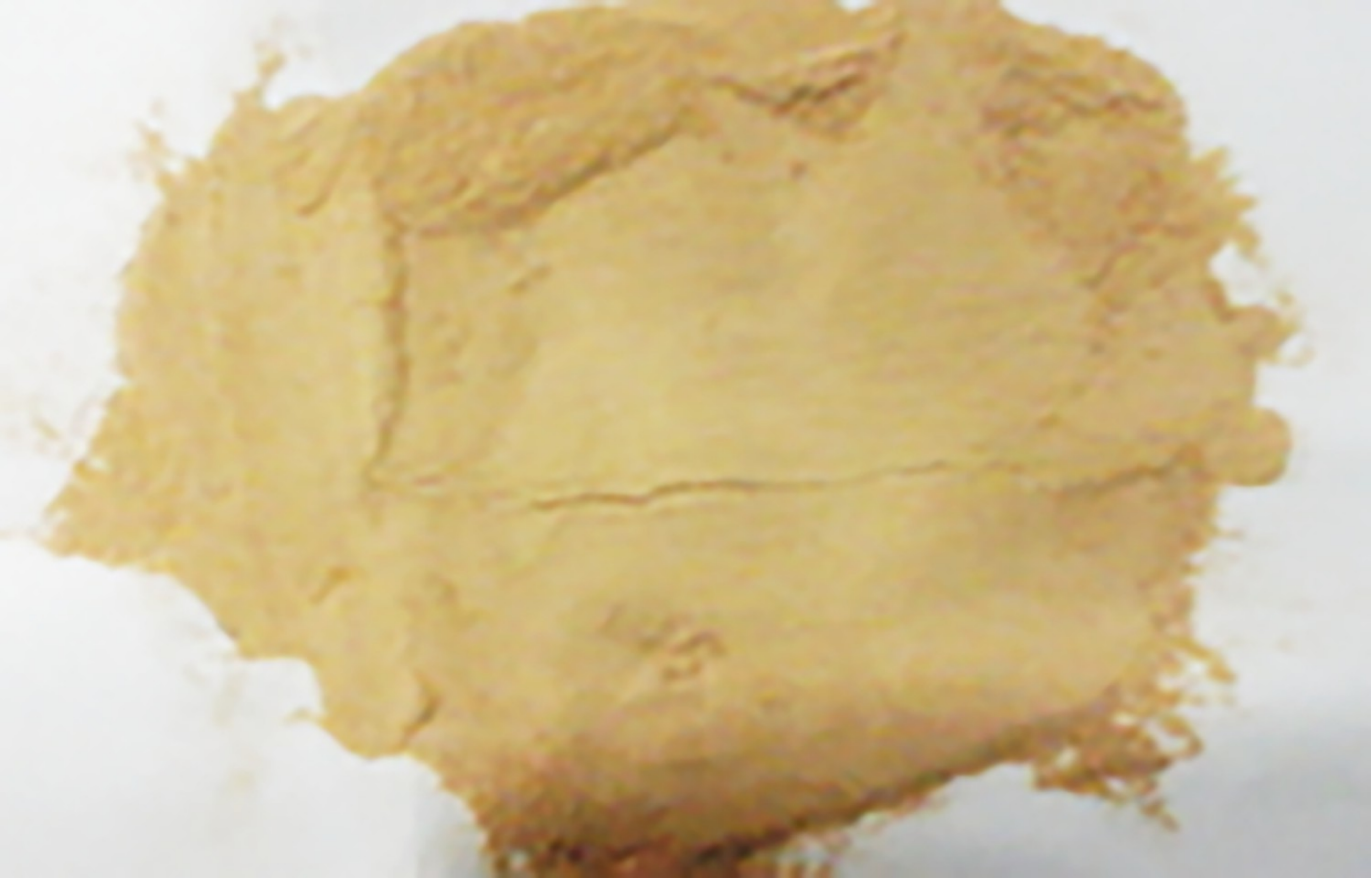
Sample B.

The sample obtained after performing the experiment as shown in Fig 5 was then characterized using different techniques to find out different characteristic properties.

#### 3.2.1. XRD

**Fig 6:**
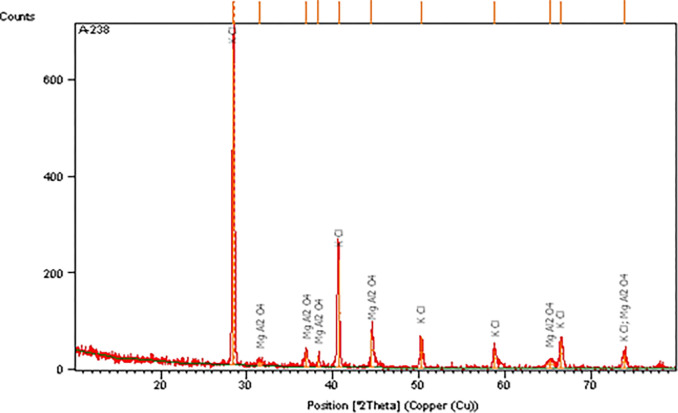
XRD result of Sample B.

Powder diffraction (XRD) is a method employed to determine the preferred orientation, crystallite size (grain size), and crystallographic structure of solid samples that are either polycrystalline or powdered. The sample needs to be homogenized, finely ground, and its average bulk composition is necessary for XRD. Magnesium aluminate spinel was examined. The parameters used in XRD for the analysis of magnesium aluminate are at ambient pressure. XRD equipment (X’pert Pro PW 3040/60, Philips, USA) using copper as the radiation source: voltage = 40 kV, current = 30 mA, no filter used, monochromator = Cu PRS (30 m). XRD detected the compounds present in the prepared sample i.e., MgAl_2_O_4_ as shown in Fig 6. An effect of the finite crystallite sizes is seen as a broadening of the peaks in an X-ray diffraction. Graph in Fig 6 representing the powder XRD patterns of prepared MgAl_2_O_4_ obtained after heat treatment of KCl precursor at 1150°C. The peaks ranging from 66° to 67° clearly show the presence of MgAl_2_O_4_ particles.

*3*.*2*.*1*.*1*. *Calculation of particle size from Scherrer equation*. Table 3 shows the particle size calculation of Sample B.

$$D=\frac{K\lambda }{\beta \;cos\theta }$$

Where,

**β** is the line broadening at half the maximum intensity (FWHM), after subtracting the instrumental line broadening, in radians.

**λ** is the X-ray wavelength,

**K** is a dimensionless shape factor, with a value close to unity. The shape factor has a typical value of about 0.9,

**D** the mean size of the ordered (crystalline) domains, which may be smaller or equal to the grain size,

**θ** is the Bragg angle (in degrees).

**Table 3:** Sample readings calculation from Scherrer Formula.

Compound	ϴ (Deg)	ϴ (Rad)	FWHM (Rad)	d-spacing (A°)	$D=\frac{K\lambda }{\beta \;cos\theta }$ (A°)	nm
KCl	14.11804	0.246	0.00183	3.14273	1545.62	154.562
KCl	14.253515	0.24877	0.00104	3.13636	2714.18	271.418
MgAl_2_O_4_	15.75469	0.274971	0.00628	2.83700	406.58	40.658
MgAl_2_O_4_	18.45143	0.322038	0.00418	2.43380	524.03	52.403
MgAl_2_O_4_	19.171395	0.334603	0.003141	2.34565	672.117	67.211
KCl	20.343935	0.355068	0.00209	2.21570	954.147	95.414
MgAl_2_O_4_	22.264215	0.388583	0.003141	2.03310	582.56	58.256
KCl	25.264215	0.438898	0.003141	1.81271	519.416	51.9416
KCl	29.399645	0.513120	0.004188	1.56916	337.22	33.722
MgAl_2_O_4_	32.613585	0.56921	0.008377	1.42920	153.55	15.355
KCl	33.23447	0.580050	0.003141	1.40548	402.737	40.273

The smallest and average crystallite size of the prepared annealed sample was determined from X‐ray line broadening using the Scherrer formula and it is found to be 15.3 nm and 46.7 nm respectively.

#### 3.2.2. Thermal analysis

**Fig 7:**
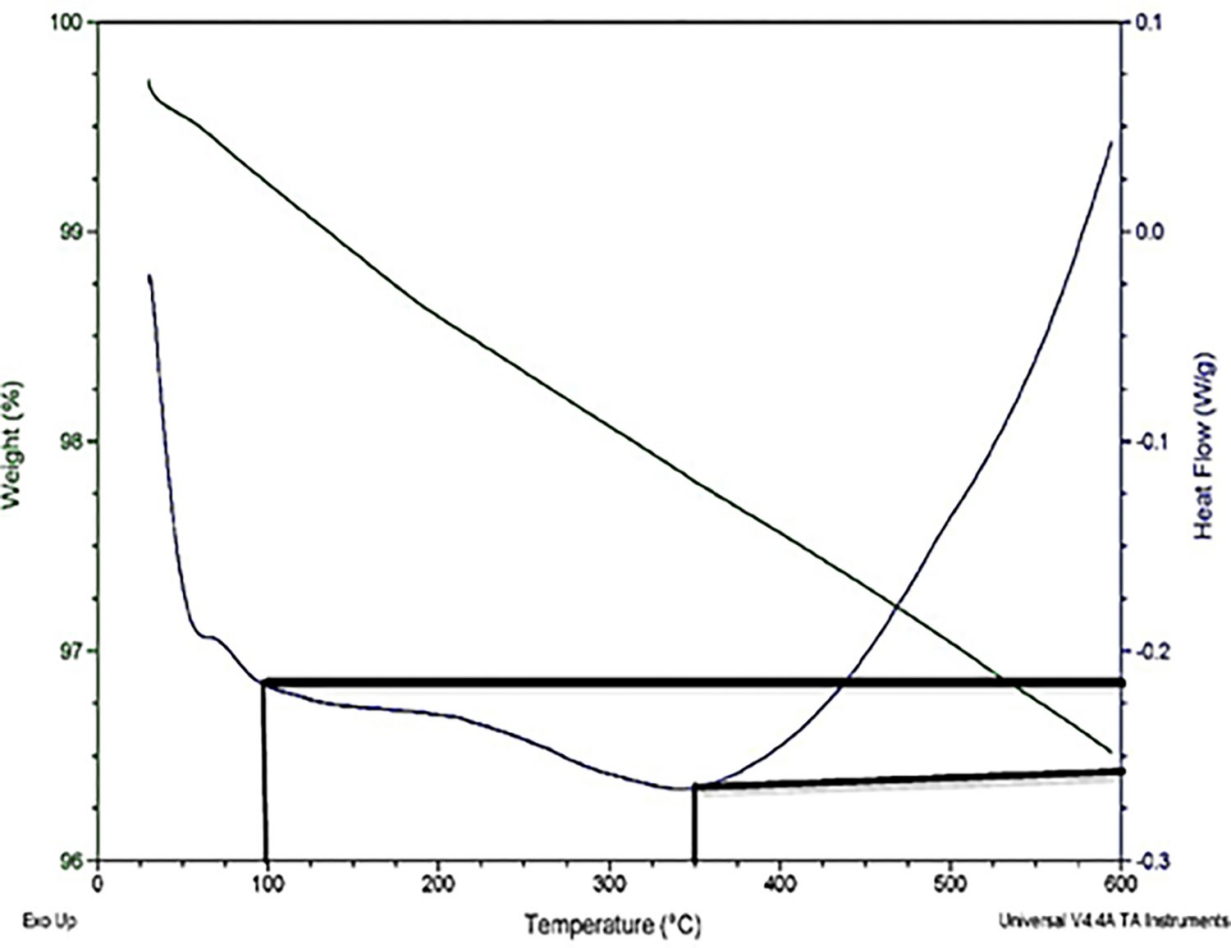
Thermal results of Sample B.

The differential scanning calorimeter (DSC) is a powerful apparatus used in the area of thermal analysis. The thermal analysis approach involves the quantification of the difference in heat energy required to increase the temperature of a specimen in comparison to a reference material. The difference is assessed as a function of temperature. DSC curve in Fig 7 shows that at 600°C, 0.05 W/g heat flows into the material and the TGA curve shows only 3.5% weight loss at 600°C. These results made MgAl_2_O_4_ more prominent for its thermal insulation application. The first hump in the curve around 100°C indicates the evaporation of moisture content. The second Hump in the curve between 250°C to 450°C showed the removal of KCl content from the sample, leaving behind pure MgAl_2_O_4_.

#### 3.2.3 Scanning electron microscopy analysis

**Fig 8:**
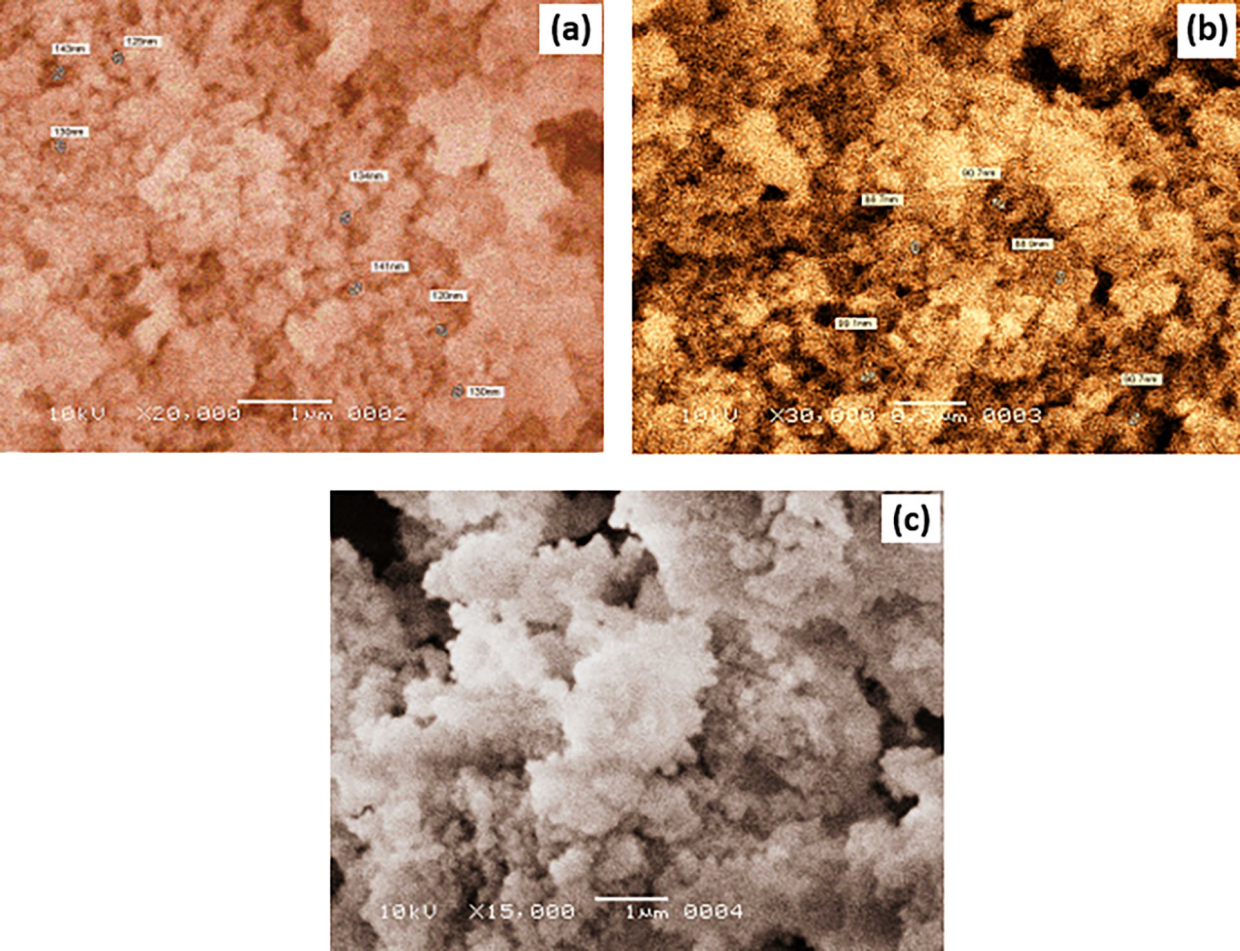
(a) SEM image at 20,000X (b) SEM image at 30,000 X (c) SEM image at 15,000 X.

The scanning electron microscope (SEM) is a variant of the electron microscope that utilizes a high-energy electron beam to scan and generate images of a sample in a raster scan pattern. JEOL Ltd. Scanning electron microscopy (SEM) is used to obtain micrographs, which confirm the presence of nanoparticles. The SEM micrograph shown in Fig 8(A) is 20,000X (particle size is in the range of 120 nm to 143 nm), but the SEM micrograph in Fig 8(B) is 30,000X (particle size is in the range of 86.7 nm to 99.1 nm), which is under nanometric scale, demonstrating the presence of irregularly shaped and sized particles. In some regions, the cohesiveness of the particles has increased, resulting in the formation of agglomerated places within the sample. Thus, it is believed that some particles within the sample are used as condensation nucleation sites around which smaller particles condense, resulting in agglomeration. Sintering of MgAl_2_O_4_ materials is challenging due to their distinct chemical stability and high melting point. The sintering properties of MgAl_2_O_4_ are influenced by the uniformity, size, and agglomeration of the particles. The irregularity and non-uniformity of the particles are attributed to the elevated calcination temperature [22–24]. Particles were agglomerated in Fig 8(A)–8(C) of SEM which was due to the high calcination temperature (i.e., 1150°C). Therefore, particles diffused into one another and connected through a physical bond. This problem can be controlled by minimizing the calcination temperature to 800°C, which is also the melting temperature of KCl. The ratio of mixed powder to solvent was also high enough that it also produced residues in sufficient quantity, and it resulted in the agglomeration of particles. If that ratio was minimized to 1:3 (i.e., mixed powder to KCl) instead of 1:30, the problem was eliminated completely.

Table 4 provides a concise overview of the synthesis and characterization of magnesium aluminate nanoparticles.

**Table 4:** Summary of synthesis and characterization of magnesium aluminate nanoparticles.

Sample	Brief overview of the obtained results
**Sample A**	**Synthesis** 6 g of each Al_2_O_3_ and MgO were mixed with 15 ml ethanol. The mixture was then placed in a ball milling and after that in a drying oven at 80°C. 2 g of the sample was mixed with 30 g of KCl. The same mixture was finally placed on a preheated graphite crucible at 1150°C. This crucible with the sample was placed in a furnace at the same temperature for 4 hours. **Characterization** The Laser Size Analyzer revealed the average size of the particle 6 μm which was beyond the nano range. Upon reviewing the experimental procedure, the main reason of the large particle size was equal mass proportion of magnesium oxide and aluminum oxide, instead of equal molar ratios.
**Sample B**	**Synthesis** 1M solutions of each MgO and Al_2_O_3_ in ethanol were prepared separately and then mixed completely. This mixture was then placed in a fume hood to get the powder of the mixture after draining out the excess ethanol. Ball milling was done of the wet mixture and dried in the oven at 80°C. 4 g of the sample was mixed with 120 g of KCl. This mixture is again milled in a mortar pestle. The same mixture was finally placed on a preheated graphite crucible at 1150°C. This crucible with the sample was placed in a furnace at the same temperature for 4 hours. **Characterization** XRD detected the presence of MgAl_2_O_4_ in a sample. The smallest and average crystallite size of the prepared annealed sample was determined from X‐ray line broadening using the Scherrer formula and it is found to be 15.3 nm and 46.7 nm respectively. DSC curve indicated 0.05 W/g heat flowed into the material at 600°C and TGA curve showed only 3.5% weight loss at the same temperature. This made MgAl_2_O_4_ good candidate for its thermal insulation application. SEM images confirmed the presence of nanoparticles with an average particle size of 91.2 nm. There was agglomeration of the particles seen due to high calcination temperature of 1150°C and high ratio of the sample i.e., 1:30. This agglomeration of the particles can be controlled by reducing the temperature up to 800°C and the sample ratio up to 1:3.

## 4. Making of crucibles

In the making of the crucible, the binder was the major component which allows the particle to bind compactly. PVA is mostly used as a binder for making the crucibles of ceramics. Table 5 shows the steps of preparing crucibles. PVA solution was made by taking PVA as a solute and water as a solvent. 5 gm of PVA powder was mixed in 100 ml of water and the mixture was stirred until all the solute particles were dissolved.

**Table 5:** List of steps for making crucibles.

Steps	Description
Mixing	Mixing of MgAl_2_O_4_ and Al_2_O_3_ with liquid polyvinyl alcohol in a desired ratio using mortar and pestle.
Transferring to Die	Transferring of resultant powder to a crucible die
Pressing	Pressing of the resultant powder in a 10-ton manual hydraulic press

### 4.1 Making of crucibles from 100% MgAl_2_O_4_

#### 4.1.1 First attempt

1g of PVA was added to 20 g of MgAl_2_O_4_ powder. The powder was then put in a mortar and pestle and mixed vigorously in order to achieve a homogenous composition. After cleaning the die and punch, oil was applied in order to avoid friction. Then the sample was placed in a die. Die and punch was placed on a hydraulic press of 10 tons. A pressure of 6 tons was applied for 5 min. The pressure was then released; the die and punch were removed from the pressing machine. The crucible was then removed from the still-attached die to the punch. The crucible was removed from the punch very carefully.

**Result:** The powder was not compressed, and it was shattered because of the low content of PVA.

#### 4.1.2 Second attempt

1.5g of PVA is added to 20 g of MgAl_2_O_4_ powder. The powder was then put in a mortar and pestle and mixed vigorously in order to achieve the homogenous composition. After cleaning the die and punch, oil was applied in order to avoid friction. Then the sample was placed in a die. Die and punch were placed on a hydraulic press of 10 tons. Pressure of 6 tons was applied for 5 min. Pressure was then released; die and punch were removed from the pressing machine. The crucible was then removed from the still-attached die to the punch. The crucible was removed from the punch very carefully.

**Result:** The compressed powder did not get the required shape when it was detached from the punch because of the low content of PVA.

#### 4.1.3 Third attempt

**Fig 9:**
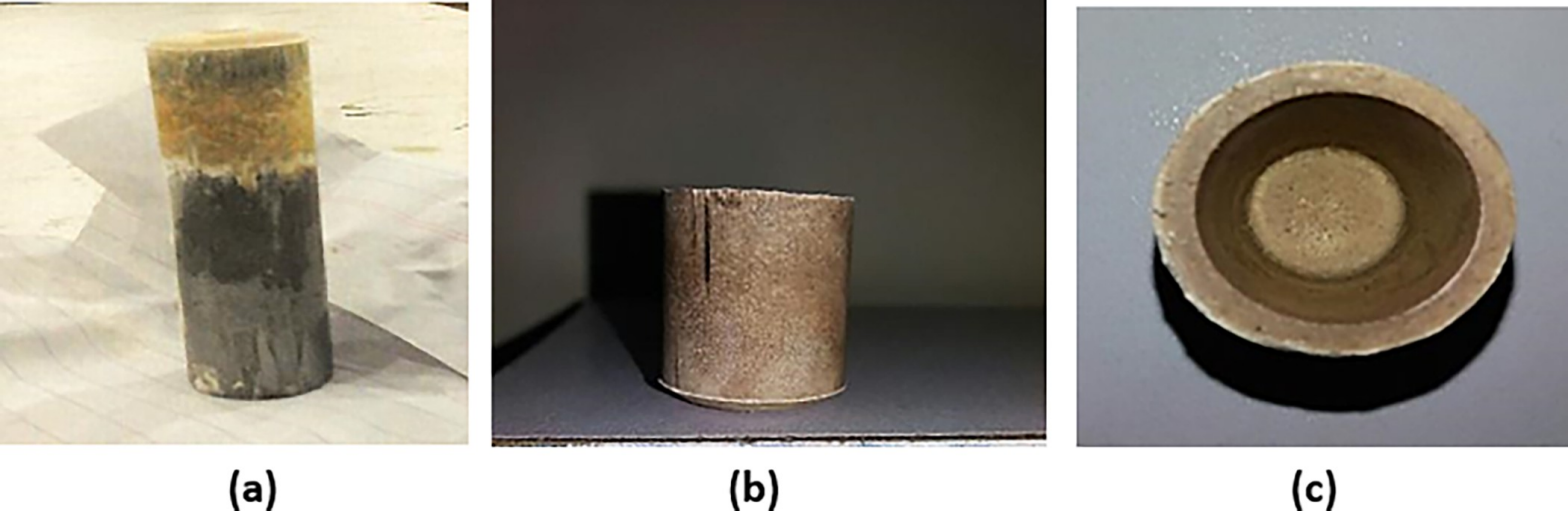
(a) Crucible attached to the punch (b) Front view of 100% MgAl_2_O_4_ unsintered crucible (c) Top view of 100% MgAl_2_O_4_ unsintered crucible.

2 g of PVA is added to 20 g of MgAl_2_O_4_ powder. The powder was then put in a mortar and pestle and mixed vigorously to achieve the homogenous composition. After cleaning the die and punch, oil was applied to avoid friction. Then the sample was placed in a die. Die and punch were placed on a hydraulic press of 10 tons. Pressure of 6 tons was applied for 5 min. Pressure was then released; die and punch were removed from the pressing machine. The crucible was then removed from the still-attached die to the punch. The crucible as demonstrated in Fig 9(A)–9(C) was removed from the punch very carefully.

### 4.2 Making of crucibles from 100% Al_2_O_3_

**Fig 10:**
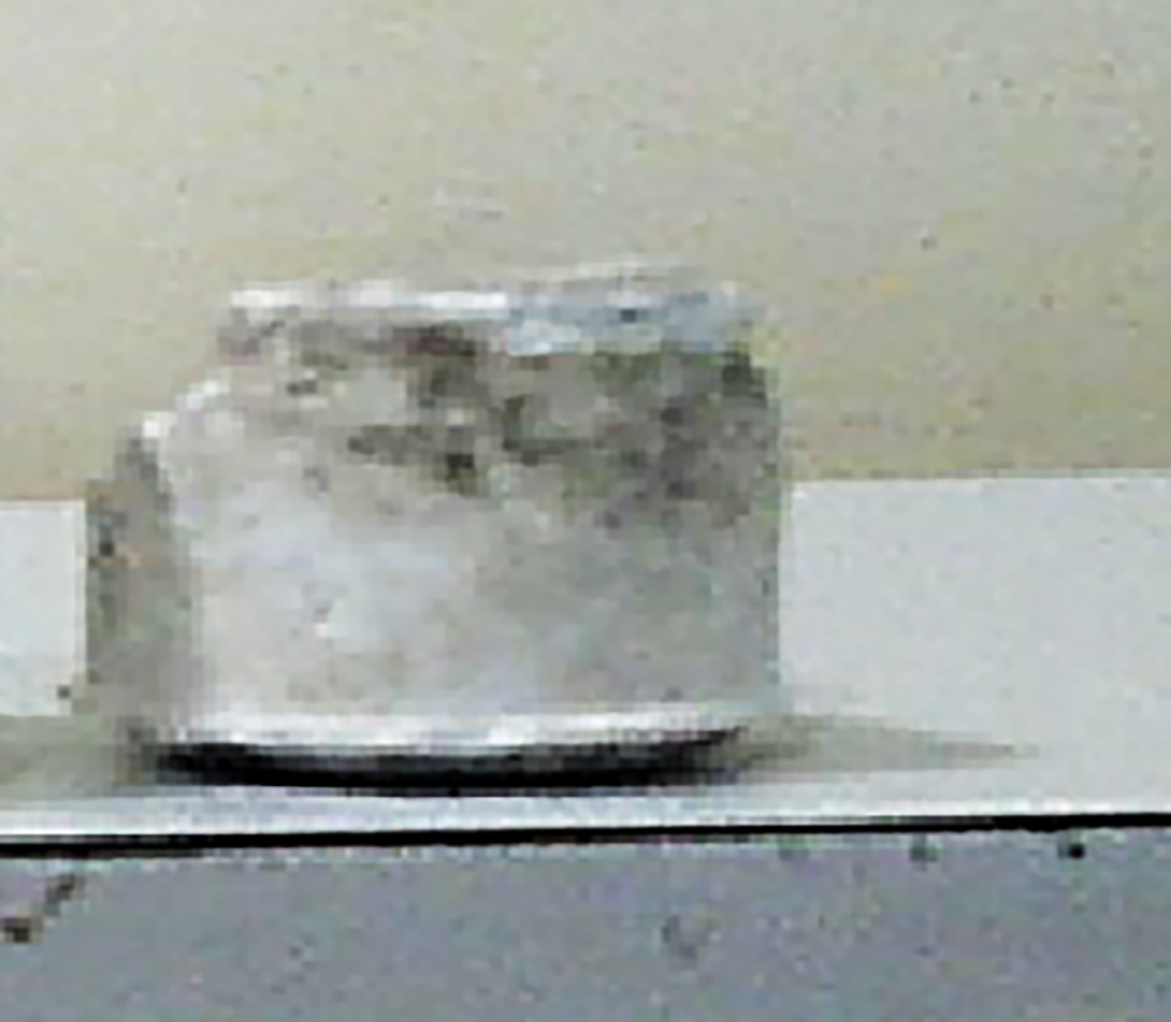
Unsintered crucible of 100% Al_2_O_3_.

2 g of PVA is added to 20 g of Al_2_O_3_ powder. The powder was then put in a mortar and pestle and mixed vigorously in order to achieve the homogenous composition. After cleaning the die and punch, oil was applied in order to avoid friction. Then the sample was placed in a die. Die and punch were placed on a hydraulic press of 10 tons. Pressure of 6 tons was applied for 5 min. The pressing machine’s die and punch were removed. The crucible was then removed from the still-attached die to the punch. The crucible as illustrated in Fig 10 was removed from the punch very carefully.

### 4.3 Making of crucibles from MgAl_2_O_4_ and Al_2_O_3_ in different ratios

#### 4.3.1 Experiment A

**Fig 11:**
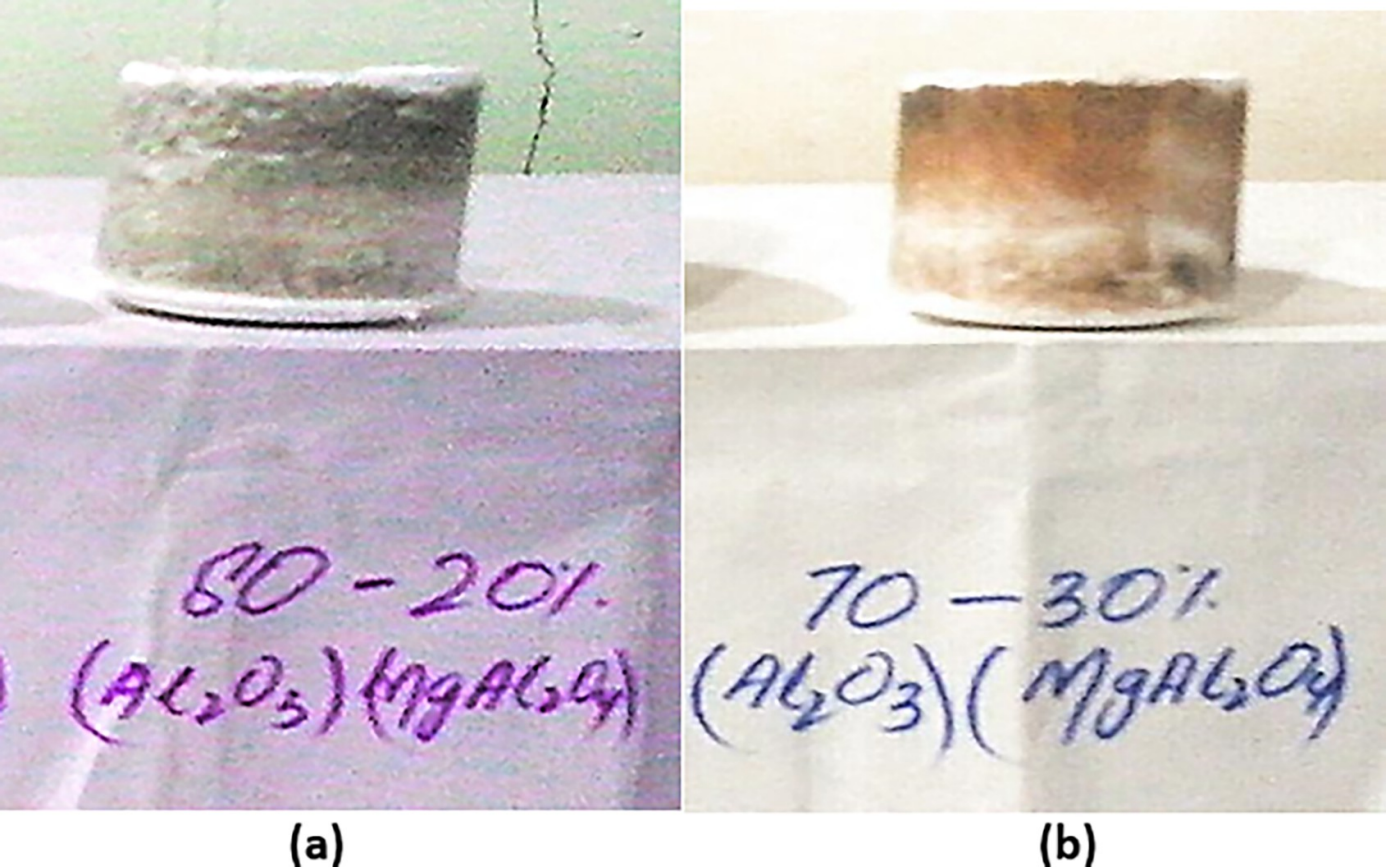
(a) Unsintered crucible of Experiment A (b) Unsintered crucible of Experiment B.

16g of Al_2_O_3_ and 4g of MgAl_2_O_4_ were placed in a mortar pestle along with 2g addition of PVA and mixed homogeneously. After cleaning the die and punch, oil was applied in order to avoid friction. Then the sample was placed in a die. Die and punch were placed on a hydraulic press of 10 tons. Pressure of 6 tons was applied for 5 min. Pressure was then released; die and punch were removed from the pressing machine. The crucible was then removed from the still-attached die to the punch. The crucible as shown in Fig 11(A) was removed from the punch very carefully.

#### 4.3.2 Experiment B

14g of Al_2_O_3_ and 6g of MgAl_2_O_4_ are placed in a mortar pestle along with 2g addition of PVA and mixed homogeneously. After cleaning the die and punch, oil was applied in order to avoid friction. Then the sample was placed in a die. Die and punch were placed on a hydraulic press of 10 tons. Pressure of 6 tons was applied for 5 min. Pressure was then released; die and punch were removed from the pressing machine. The crucible was then removed from the still-attached die to the punch. The crucible as shown in Fig 11(B) was removed from the punch very carefully.

A summary of different compositions used in Experiment A and Experiment B is shown in Table 6.

**Table 6:** Different composition of crucibles.

Experiment	Composition (MgAl_2_O_4_, Al_2_O_3_)	MgAl_2_O_4_	Al_2_O_3_
A	(20%, 80%)	4g	16g
B	(30%, 70%)	6g	14g

**Fig 12:**
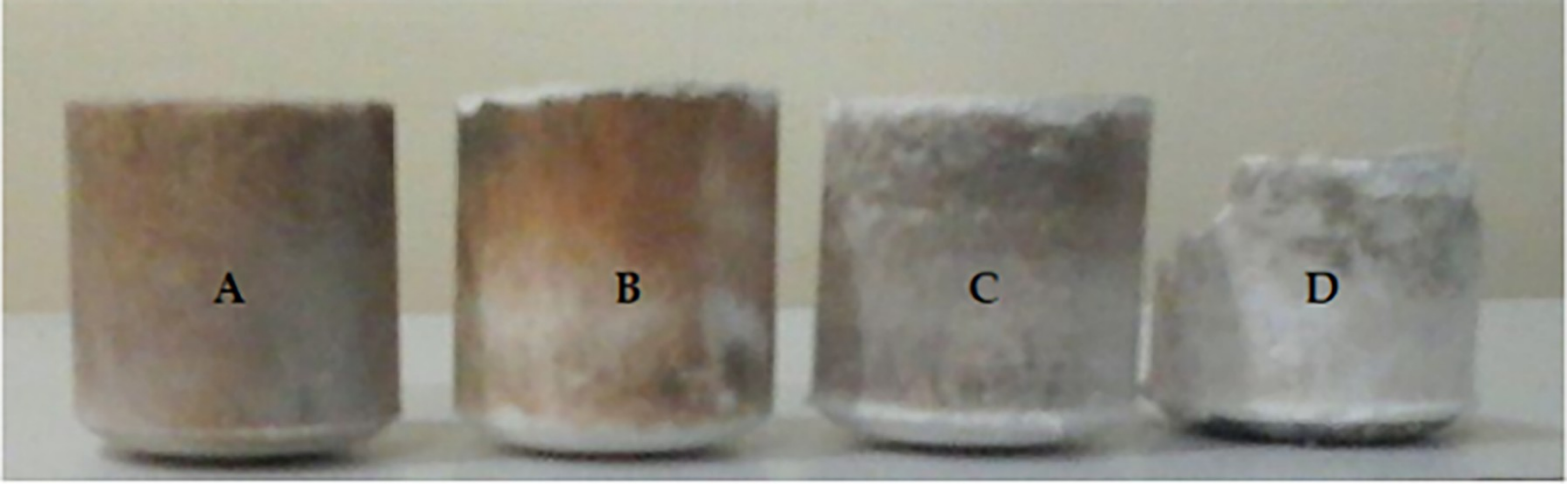
Unsintered crucibles (A) 100% MgAl_2_O_4_ (B) 80% Al_2_O_3_ and 20% MgAl_2_O_4_ (C) 70% Al_2_O_3_ and 30% MgAl_2_O_4_ (D) 100% Al_2_O_3_.

All four unsintered crucibles are depicted in Fig 12.

### 4.4 Sintering of crucibles

**Fig 13:**
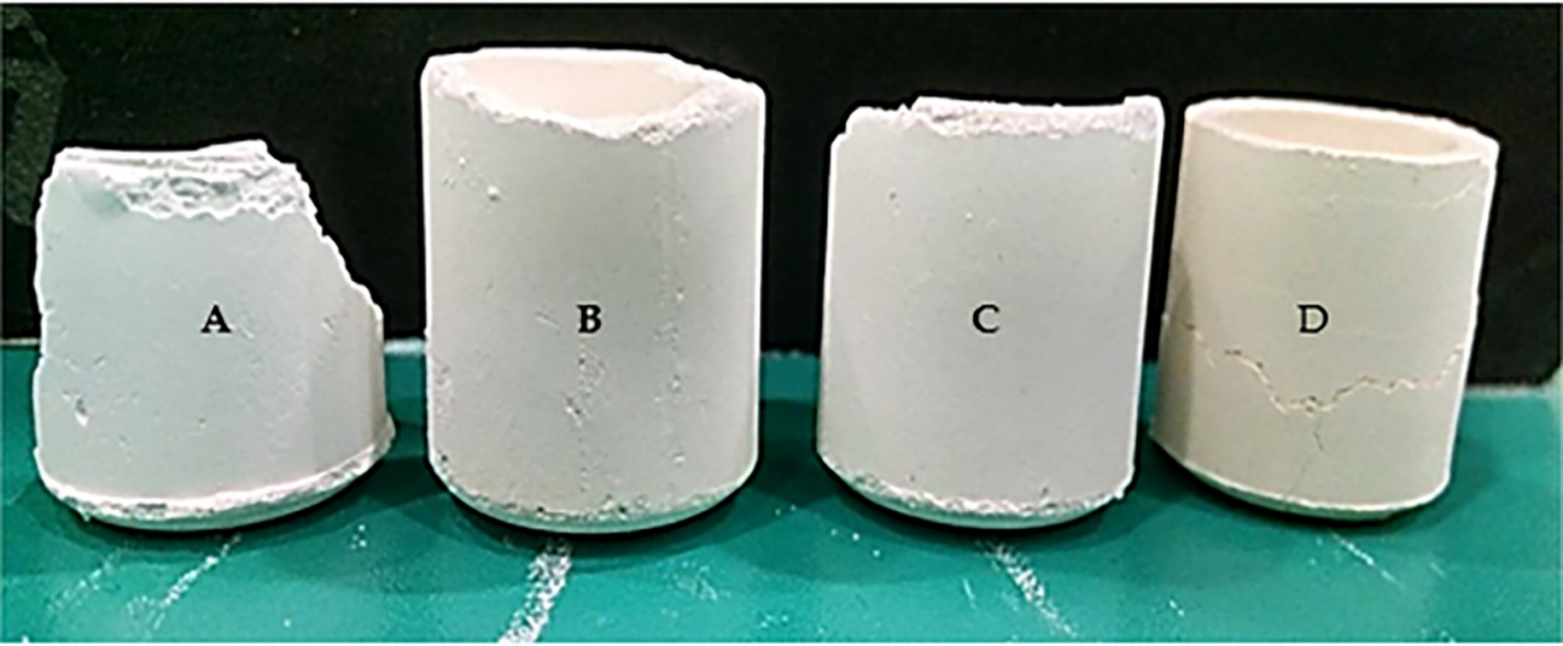
Sintered crucibles (A) 100% MgAl_2_O_4_ (B) 80% Al_2_O_3_ and 20% MgAl_2_O_4_ (C) 70% Al_2_O_3_ and 30% MgAl_2_O_4_ (D) 100% Al_2_O_3_.

Sintering of all four crucibles (i.e., 100% MgAl_2_O_4_, 80% Al_2_O_3_ and 20% MgAl_2_O_4_, 70% Al_2_O_3_ and 30% MgAl_2_O_4_, 100% Al_2_O_3_) were conducted at 1100°C for 2 hours along with 30 min holding at 400°C and 30 min holding at 700°C. The resulting crucible was then furnace cooled as shown in Fig 13. Three crucibles were successfully sintered without any damage, however the sintering process for 100% MgAl_2_O_4_ did not yield satisfactory results. Hence, a comprehensive examination into the phenomenon of cracking is carried out.

### 4.5 Making of tablets to carry out the investigation of sintering of 100% MgAl_2_O_4_

Tablets of 100% MgAl_2_O_4_ were made to study the effects of sintering and densification of particles as the crucibles made in the experiment did not seem to be sintered at the described conditions.

#### 4.5.1 Preparation of tablets

1g of PVA was added to 10g of the sample and mixed vigorously till complete homogenization. Then the powder was divided into three equal quantities. Three tablets were made for the confirmation of tests. The tablets were made with a 1cm diameter die and punch. A pressure of 5000 psi was applied to compact the powder. The densities of tablets were calculated after compaction by measuring the height and diameter of the tablets with a vernier caliper. The green tablets were sintered at 1150°C for 2 hours, followed by 30 minutes at 300°C and 30 minutes at 700°C. The densities of tablets were calculated after sintering by using the same procedure as mentioned above. Stereographic images were also taken before and after the sintering of tablets.

For Ceramography, samples were hot mounted. Grinding of samples was done using 400 grit size of silicon carbide paper for 2 min, followed by grinding on 600 grit size paper for 2 min and finally on 1000 grit size paper for 2 min. The samples were washed and polished using coarse alumina powder (i.e., 1μm) for 2 min, followed by polishing using fine alumina powder (i.e., 0.5 μm). The samples were washed once more before being etched for 10 minutes with concentrated Aqua regia and hot blowing for 3 minutes. The samples were then taken to the Olympus Multicolor Microscope and images were taken at 400 X.

#### 4.5.2 Density of tablets

The density of any material can be affected by two parameters (i.e., mass and volume). During sintering, both parameters may be affected. Loss of PVA and moisture content results in weight loss, and shrinkage of geometry results in volume decreases. However, a marginal change in density was observed due to both of the above-mentioned reasons. Table 7 shows the density of tables before and after sintering.

**Table 7:** Calculations of density before and after sintering.

S.No	Parameters	Unsintered	$\left(\rho =\frac{m}{\pi r^{2}h}\right)$	Sintered	$\left(\rho =\frac{m}{\pi r^{2}h}\right)$
1	Height (h)	0.71cm		0.71cm	
2	Radius (r)	0.6375cm	1.864 g/cm^3^	0.625cm	1.916 g/cm^3^
3	Mass (m)	1.69g		1.67g	

#### 4.5.3 Stereo micrographs

**Fig 14:**
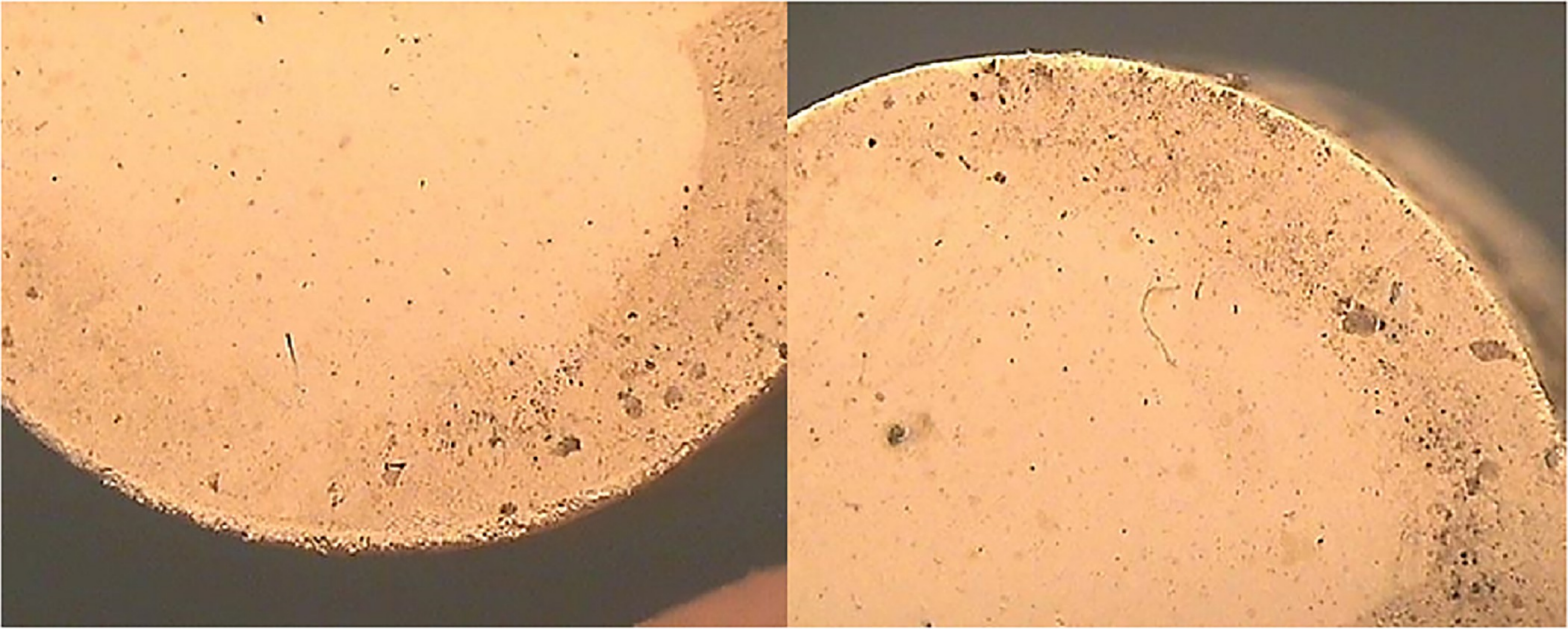
Stereographic images of unsintered tablets at 15X.

Stereographic images were taken at 15X of tablets as shown in Fig 14. Before sintering, the blackish area on the surface of the sample indicated the oil content which was applied during compression of the tablet. Moreover, the metal particle inclusions were embedded on the sample from the die which can be seen as tiny sharp black spots. In addition, the tablets’ edges were damaged during their removal from the die.

**Fig 15:**
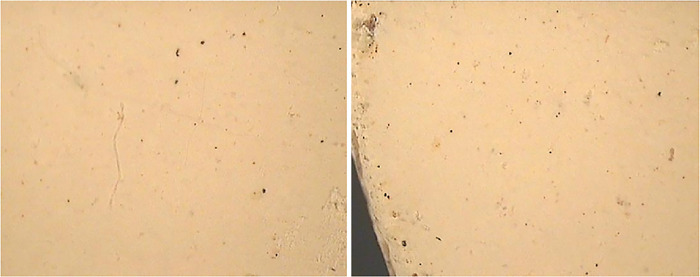
Stereographic images of sintered tablets at 15X.

After sintering, the surface of the sample appeared to be clearer and brighter due to the removal of oil content. The particles were well densified and compacted and other inclusions like black spots were also minimized due to the sintering as shown in Fig 15.

#### 4.5.4 Ceramography

**Fig 16:**
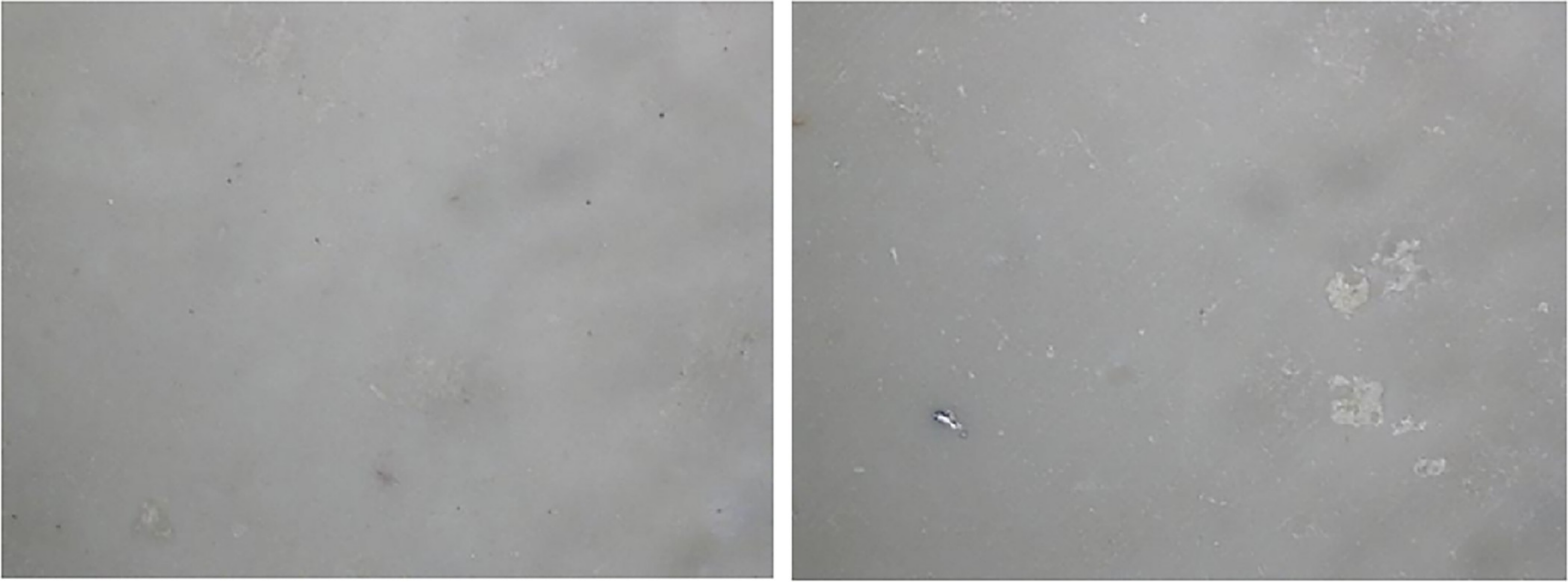
Micrographs of sintered sample at 400X.

Images of the surfaces of sintered tablets were taken by using a metallurgical microscope at 400X as demonstrated in Fig 16.

The surfaces were found to be dull and clear. It has a compact and dense structure and was unaffected by the etchant used. White spots on the surface were due to the alumina which was embedded on the surface during polishing. Sintering was achieved at 1150°C, which could be seen from the micrographs. Compactness can be enhanced by increasing the sintering temperature and the holding time.

#### 4.5.5 Hardness of sintered sample

**Fig 17:**
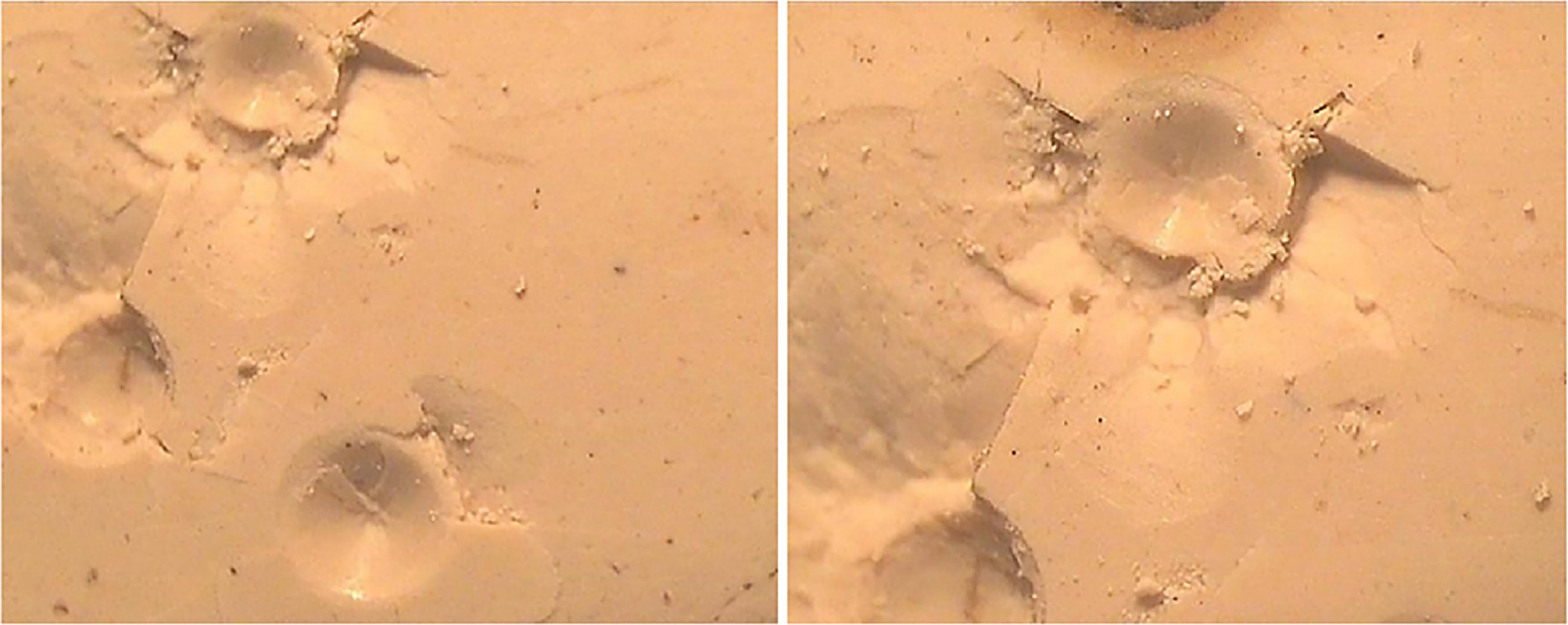
Stereographic images of indented sample at 15X.

Hardness was taken by Rockwell hardness tester. Steel ball indenter applied 100 kg of force for a dual time of 10 seconds. These indents were taken close to each other. Stereographic images showed that very less damage occurred on the surface. During testing, a crack developed in the mounting area, but the sample remained intact, and the hardness of the sample was estimated to be above the measuring scale. Ball penetrated less than 1mm into the surface which can be seen in the images as illustrated in Fig 17.

## 5. Conclusion

The molten salt technique was employed in order to obtain nanoparticles of MgAl_2_O_4_. Potassium chloride (KCl) was employed as the solvent in the experiment. The laser particle analyzer was utilized to characterize Sample A, revealing that the average particle size measured 6 μm, exceeding the nano range. It is evident that the primary cause for the significant particle size was the equimass proportion of magnesium oxide and aluminum oxide, as opposed to equimolar ratios. Consequently, a sample denoted as B has been meticulously created by combining equal molar amounts of magnesium oxide (MgO) and aluminum oxide (Al_2_O_3_) in an ethanol solvent. The confirmation of MgAl_2_O_4_ nanoparticles derived from Sample B was achieved by the analysis of X-ray diffraction (XRD) findings. The observed peaks within the range of 66° to 67° provide unambiguous evidence of the existence of MgAl_2_O_4_ particles. The determination of the smallest and average crystallite size of the sample was achieved by applying the Scherrer formula. The resulting measurement revealed a value of 15.3 nm and 46.7 nm respectively. The scanning electron microscope (SEM) images also presented evidence of an average particle size of 91.2 nm. The differential scanning calorimetry (DSC) analysis reveals that at a temperature of 600°C, the material experiences a heat flow of 0.05 W/g. Additionally, the thermogravimetric analysis (TGA) curve demonstrates a weight loss of only 3% at 600°C. This characteristic is particularly significant when considering the material’s potential application in thermal insulation. Ultimately, crucibles were fabricated with a combination of MgAl_2_O_4_ and high-purity alumina. Additionally, these substances were combined in varying proportions to create diverse compositions of crucibles. Sintering of all four crucibles (i.e., 100% MgAl_2_O_4_, 80% Al_2_O_3_ and 20% MgAl_2_O_4_, 70% Al_2_O_3_ and 30% MgAl_2_O_4_, and 100% Al_2_O_3_) was conducted at 1100°C. However, cracks appeared on the crucible composed of 100% magnesium aluminate during the sintering process. Therefore, a comprehensive examination of the phenomenon of cracking is carried out at 1150°C by making the samples in the form of small tablets of damaged crucible. The tablets undergo ceramography and hardness testing in order to examine the reason for crack development. The results revealed that the absence of cracks on the surfaces of all three tablets shows that a minimum temperature of 1150°C was necessary for the sintering process of MgAl_2_O_4_. Future studies should examine the investigation of incorporating MgAl_2_O_4_ into Al_2_O_3_ crucibles, which would enhance the characteristics of the material, including its strength at elevated temperatures and thermal insulating properties. The enhancement of the mechanical properties of a magnesium aluminate crucible would also be achieved by the utilization of elevated sintering temperatures and extended furnace holding times. The rate at which the furnace generates heat also has an impact on the sintering process. By utilizing the lowest heating rate, it is possible to enhance the strength of the crucible as well.

## References

[1] Almajid A, Walter R, Kroos T, Junaedi H, Gurka M, Khalil KA. The multiple uses of polypropylene/polyethylene terephthalate microfibrillar composite structures to support waste management—composite processing and properties. Polymers. 2021;13(8):1296. doi: 10.3390/polym13081296 33921061 PMC8071395

[2] Almajid A, Walter R, Kroos T, Junaidi H, Gurka M, Abdelrazek Khalil K. Development of Polypropylene/Polyethylene Terephthalate Microfibrillar Composites Filament to Support Waste Management. Polymers. 2021, 13, 233. doi: 10.3390/polym13020233 33445424 PMC7827194

[3] Almajid AA. High-temperature deformation of naturally aged 7010 aluminum alloy. Metals. 2021;11(4):581.

[4] Nassar MY, Ahmed IS, Samir I. A novel synthetic route for magnesium aluminate (MgAl_2_O_4_) nanoparticles using sol–gel auto combustion method and their photocatalytic properties. Spectrochim Acta A Mol Biomol Spectrosc. 2014;131:329–34. 24835935 10.1016/j.saa.2014.04.040

[5] Rahmat N, Yaakob Z, Pudukudy M, Rahman NA, Jahaya SS. Single step solid-state fusion for MgAl_2_O_4_ spinel synthesis and its influence on the structural and textural properties. Powder Technol. 2018;329:409–19.

[6] Shahid F, Ismail B, Khan AM, Ain QU, Khan RA, Shah F, et al. Cost effective way of tuning physical properties of MgAl_2_O_4_spinel nanomaterials by Sr+ 2/Mn2+ cations doped at the T-Sites. Ceram Int. 2020;46(8):10710–7.

[7] Varghese P, Shukla PK, Vetrivendan E, Das SK, Ponraju D, Ningshen S. Long-term exposure of MgAl_2_O_4_ and Y2O3 thermal barrier coatings in molten sodium. Surf Coat Technol. 2020;381:125111.

[8] Ganesh I, Reddy GJ, Sundararajan G, Olhero SM, Torres PM, Ferreira JM. Influence of processing route on microstructure and mechanical properties of MgAl_2_O_4_ spinel. Ceram Int. 2010;36(2):473–82.

[9] Takahashi S, Kan A, Ogawa H. Microwave dielectric properties and crystal structures of spinel-structured MgAl2O4 ceramics synthesized by a molten-salt method. J Eur Ceram Soc. 2017;37(3):1001–6.

[10] Ganesh I. A review on magnesium aluminate (MgAl_2_O_4_) spinel: synthesis, processing and applications. Int Mater Rev. 2013;58(2):63–112.

[11] Safaei-Naeini Y, Aminzare M, Golestani-Fard F. The effects of temperature and different precursors in the synthesis of nano spinel in KCl molten salt. Ceram Int. 2012;38(1):841–5.

[12] Fazli R, Fazli M, Safaei-Naeini Y, Golestani-Fard F. The effects of processing parameters on formation of nano-spinel (MgAl_2_O_4_) from LiCl moltensalt. Ceram Int. 2013;39(6):6265–70.

[13] Zhang DL, Li BR, Hu YH, Li JT, Guo YQ. Synthesis and characterization of MgAl_2_O_4_ micro-rods by a molten salt method. Ceram Int. 2015;41(4):5881–7.

[14] Zhang J, Li D, Zhou Y. Molten salt synthesis of magnesium aluminate (MgAl_2_O_4_) spinel on Ti_3_AlC_2_ substrate. J Am Ceram Soc. 2009;92(5):1074–8.

[15] Sinhamahapatra S, Das P, Dana K, Tripathi HS. Magnesium Aluminate Spinel: Structure, Properties, Synthesis and Applications. Trans. Indian Ceram. Soc. 2022;81(3):97–120.

[16] Fujii K, Furusato I, Takita I. Composition of spinel clinker for teeming ladle casting materials. Taikabutsu Overseas. 1992;12(1):4–9.

[17] Yamamura T, Hamazaki Y, Kaneshige T, Toyoda T, Nishi M, Kato H. Alumina-spinel castable refractories for steel teeming ladle. Taikabutsu Overseas. 1992;12(1):21–7.

[18] Jeong DH, Lee SK. Development of carbon contained MgO-spinel castables for teeming ladle. RIST J. R&D. 2005;19(3):197–206.

[19] Dal Maschio R, Fabbri B, Fiori C. Industrial applications of refractories containing magnesium aluminate spinel. Ind. Ceram. 1988;8(3):121–6.

[20] Tokunaga K, Kozuka H, Honda T, Tanemura F. Further improvement in high temperature strength, coating adherence, and corrosion resistance of magnesia-spinel bricks for rotary cement kiln. InUnitecr’91. Unified Int. Technical Conf. Refractories. Global Advances in Refractories. Preprints. Aachen 1991; 23(26).

[21] Mouyane M, Jaber B, Bendjemil B, Bernard J, Houivet D, Noudem JG. Sintering behavior of magnesium aluminate spinel MgAl2O4 synthesized by different methods. Int. J. Appl. Ceram. Technol. 2019;16(3):1138–49.

[22] Yan Z, Wang H, Yu J. Study on Sinterability of Magnesium Aluminate Spinel Powders Prepared by Different Technologies. IOP Conf Ser Mater Sci Eng 2019; 678(1):012011.

[23] Figueredo GP, Carvalho AF, Medeiros RL, Silva FM, Macêdo HP, Melo MA, et al. Synthesis of MgAl 2 O 4 by gelatin method: effect of temperature and time of calcination in crystalline structure. Mater. Res. 2017; 20:254–9.

[24] Fu P, Lu W, Lei W, Wu K, Xu Y, Wu J. Thermal stability and microstructure characterization of MgAl2O4 nanoparticles synthesized by reverse microemulsion method. Mater. Res. 2013;16:844–9.

